# Identification of Germline Mutations in Upper Tract Urothelial Carcinoma With Suspected Lynch Syndrome

**DOI:** 10.3389/fonc.2022.774202

**Published:** 2022-03-16

**Authors:** Bao Guan, Jie Wang, Xuesong Li, Lin Lin, Dong Fang, Wenwen Kong, Chuangyu Tian, Juan Li, Kunlin Yang, Guanpeng Han, Yucai Wu, Yuhui He, Yiji Peng, Yanfei Yu, Qun He, Shiming He, Yanqing Gong, Liqun Zhou, Qi Tang

**Affiliations:** ^1^ Department of Urology, Peking University First Hospital, Beijing, China; ^2^ Institute of Urology, Peking University, Beijing, China; ^3^ National Urological Cancer Center, Beijing, China; ^4^ Department of Anorectal, Yantai Baishi Anorectal Hospital, Yantai, China; ^5^ Key Laboratory of Genomics and Precision Medicine, Beijing Institute of Genomics, Chinese Academy of Sciences, Beijing, China

**Keywords:** DNA mismatch repair, upper tract urothelial carcinoma, inherited cancer, Lynch syndrome, whole-exon sequence

## Abstract

**Objective:**

Whole-exon sequencing (WES) is a commercially available tool for hereditary disease testing. However, little is known about hereditary upper-tract urothelial carcinoma (UTUC) in the Chinese population. This study aims to investigate the prevalence of Lynch syndrome (LS) in UTUC patients with high-risk features and identify the germline mutations of genetic predisposition gene mutations in those patients.

**Methods:**

In total, 354 consecutive UTUC patients undergoing surgery were universally recruited, of whom 108 patients under 60 years old or with a personal/family history of cancer underwent universal immunohistochemistry staining to detect the expression of mismatch repair (MMR) proteins (MLH1, MSH2, MSH6 and PMS2). Patients with deficient or weak MMR protein staining or meeting the Amsterdam II criterion were defined as suspected LS patients, who further experienced microsatellite instability (MSI) (BAT25, BAT26, BAT40, D2S123, D5S346, D17S250) detection and performed WES analysis to explore germline pathogenic/likely pathogenic (P/LP) alterations.

**Results:**

Of 108 patients, 90 (83.3%) cases were included due to younger than 60 years, and 18 cases due to personal/family history. IHC staining identified 21 patients with deficient MMR protein staining and 15 cases with weak MMR protein staining. Three cases met the Amsterdam II criterion but with proficient MMR protein staining. Finally, WES analysis was performed in 38 suspected LS patients and P/LP germline mutations were identified in 22 individuals. Genetic testing confirmed 5 LS cases, including 3 cases with novel mutations. MSI-harboring tumor was discovered in 4 LS cases, one of whom had weak MMR protein staining. Germline P/LP variants in DNA damage repair genes were found in 11 cases. In addition, we found that 11 patients had high- or moderate- penetrance P/LP mutations other than MMR genes. The common P/LP variants in high- or moderate-penetrance genes were 4 in ATM, 3 in MSH6 and KIT, and 2 in APC, NF1 and DICER.

**Conclusions:**

We identified approximately 11% of UTUC cases as suspected LS and at least 1.4% patients with confirmed LS-associated UTUC. In addition, broader germline genetic testing could be considered to screen for cancer severity in hereditary UTUC patients.

## Introduction

Hereditary diseases are typically correlated with an advanced risk of various symptoms or malignancies. Determining a specific genetic cancer susceptibility is essential to provide patients and their families with an opportunity for disease surveillance and potential guidance for preventive treatment. One of the most prevalent hereditary malignancies is Lynch syndrome (LS), which results from pathogenic alterations in one of the mismatch repair (MMR) genes.

As an autosomal dominant genetic susceptibility syndrome, LS is prone to early-onset colorectal cancer and other related cancers, especially upper tract urothelial carcinoma (UTUC) (1). The approximate incidence rate of UTUC in individuals with LS ranges from 1% to 28% (2). Routine screening of LS-related UTUC has recently been brought into clinical practice for a decade, yet current studies have reported that the prevalence of LS in UTUC is 3%-21% (3–7). The classical inherited cancer risk evaluation method includes identifying subjects whose medical history meets the clinical diagnostic standard for a particular genetic disease and then performing targeted sequencing only on the genes related to the disease (8). Although clinical criteria and prediction tools can help guide genetic testing for LS, approximately 30% to 50% of families who meet strict clinical diagnostic standards for LS will eventually fail to detect pathogenic germline MMR gene mutations (9). In addition, it is pyramidally recognized that the broad phenotypic extent of LS-related tumors may overlap with other inherited diseases (9). Therefore, the traditional diagnostic standard is probably not the ideal inherited disease hazard evaluation tactic in suspected LS patients.

Next-generation sequencing (NGS) technology can analyze a large number of genetic susceptibility genes simultaneously, possessing the strength of high efficiency and affordability, and is convenient for use in exploring other potential genetic diseases (8, 10). However, few studies on LS-related UTUC identified by genetic testing have been reported, and whole exon sequencing (WES) analysis of germline DNA has also been scarce. Thus, we proposed a selective screening process for LS in high-risk UTUC patients using immunohistochemistry (IHC), followed by microsatellite instability (MSI) analysis andWES, and further identified the frequency of genetic predisposition gene mutations among suspected LS patients.

## Materials and Methods

### Patient Selection

**Figure 1:**
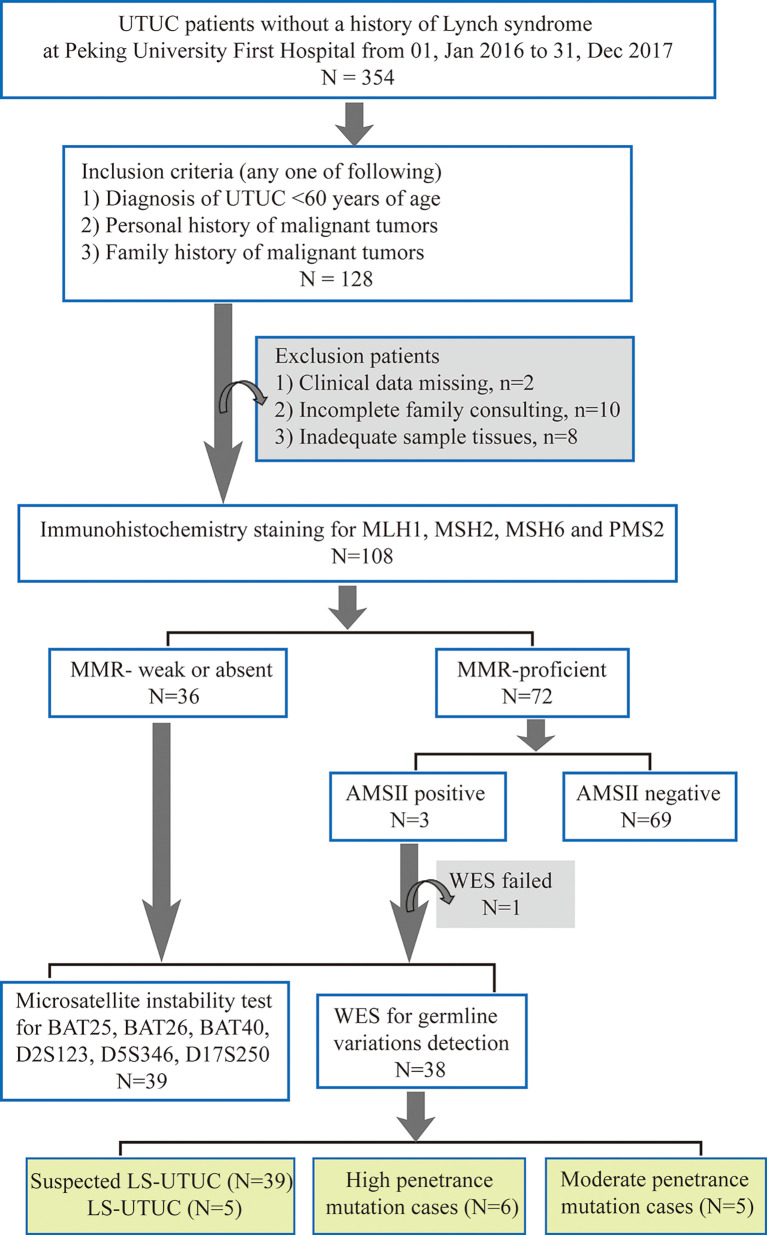
Flowchart of Lynch Syndrome screening. UTUC, upper tract urothelial carcinoma; MMR, mismatch repair; AMS, Amsterdam; WES, whole exon sequencing; LS-UTUC, Lynch Syndrome related UTUC.

In total, 354 consecutive UTUCs without a history of LS undergoing surgery from Peking University First Hospital between Jan 01, 2016 and Dec 31, 2017 were collected. The patients were selected according to three inclusion criteria (one of any): 1) diagnosis of UTUC <60 years of age; 2) personal history of malignant tumors; and 3) family history of malignant tumors. Finally, 108 patients who met above criteria were performed MMR protein staining. The flowchart of patient selection was showed in **Figure 1**. Personal/Family history information is collected from medical archives and during follow-up. The LS-associated tumor includes colorectal cancer, endometrial cancer, intestine cancer, gastric cancer, pancreatic cancer, ovarian cancer, urothelial carcinoma, biliary tract carcinoma, sebaceous adenomas/cancer, and cerebral tumors. This study was approved by the Peking University First Hospital Ethics Committee. All patient-derived samples and clinicopathological information were collected after verbal informed consent was obtained.

### Immunohistochemical Staining

Immunohistochemical staining of the MMR proteins MLH1, MSH2, MSH6 and PMS2 was performed on paraffin-embedded tissue sections of 108 patients (**Supplementary Material**). Four μm thick FFPE tissue sections were stained with primary monoclonal antibodies against MSH2 (Abcam, UK, ab52266, mouse), MSH6 (Abcam, UK, ab92471, rabbit), MLH1 (Abcam, UK, ab92312, rabbit), and PMS2 (Abcam, UK, ab110638, rabbit). The proficient expression of MMR proteins in tumor cells was determined by the presence of nuclear staining. Loss of staining in cancer cells with positive nuclei in the positive control in parallel indicates a loss of protein expression (**Supplementary Figure 1**). Weak protein staining was defined by the absence of nuclear staining in more than half of urothelial carcinoma cells and weak staining of the remaining tumor cells (**Supplementary Figure 2**). The processed IHC slides were blindly evaluated by 2 pathologists.

### MSI Analysis

DNA from 39 suspected LS patients, including 36 patients with deficient or weak MMR protein staining and 3 patients who met Amsterdam II criteria, were extracted (**Supplementary Methods**). A panel of 6 microsatellites (BAT25, BAT26, BAT40, D2S123, D5S346, and D17S250) was used to determine the MSI status of 39 matched samples. We defined the MSI status as stable (no allele altered), low (1 allele altered) and high (≥2 altered markers).

### Germline Sequencing and Interpretation

Because the tumor from a patient meeting Amsterdam II criteria was unable to build library and perform WES, whole exon sequencing analysis were only carried out on 38 patients with suspected LS (**Supplementary Methods**). The genes analyzed in this study were summarized in **Supplementary Table 1**. Pathogenicity was identified in accordance with the American College of Medical Genetics standard (11). Alteration types in inherited susceptible genes were judged by PathoMAN (https://pathoman.mskcc.org/) (10, 12), which is an automated hereditary mutation evaluation software. Consistent with prior studies (10, 13), all sequence variants were classified into the following tiers: pathogenic, likely pathogenic, uncertain clinical significance, likely benign, benign, and polymorphism.

### Statistical Analysis

Parameters were compared by the chi-squared test, Fisher’s exact test, Wilcoxon rank test or Kruskal-Wallis test as indicated. P-values < 0.05 were considered statistically significant, and all tests were two-sided. All statistical analyses were performed in SPSS version 15.0 (IBM Corporation, America).

## Results

### Patient Characteristics

Ninety patients (83.3%) were recruited due to ages younger than 60 years, and 18 patients (16.7%) were recruited due to personal or family history of malignant tumors (**Supplementary Table 2**). The median age of 108 patients was 55 years (range, 29-84 years), and 47 patients (43.5%) were female. IHC staining showed 21 (19.4%) patients with deficient MMR protein staining and 15 patients (13.9%) with MMR proteins weak staining. In total, 12 patients were deficient MSH6 or MSH6/MSH2 protein staining, while 7 patients had deficient MLH1 protein staining and no patient had deficient PMS2 protein staining(**Supplementary Table 2**). Suspected LS patients were significantly associated with no history of bladder cancer or concurrent bladder cancer, but were not associated with gender, pathological variables or the presence of personal or first-degree relatives LS-related cancer (**Supplementary Table 3**).

### Germline Findings

**Figure 2:**
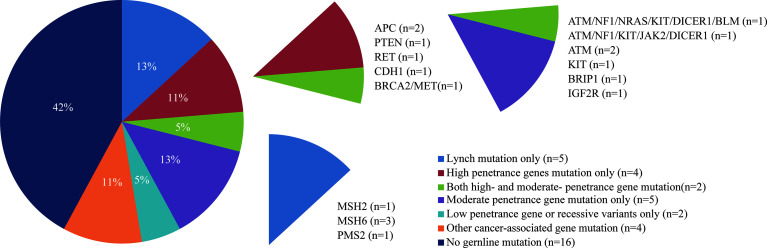
Frequency and penetrance of germline pathogenic/likely pathogenic variants in patients with suspected LS-UTUC.

**Figure 3:**
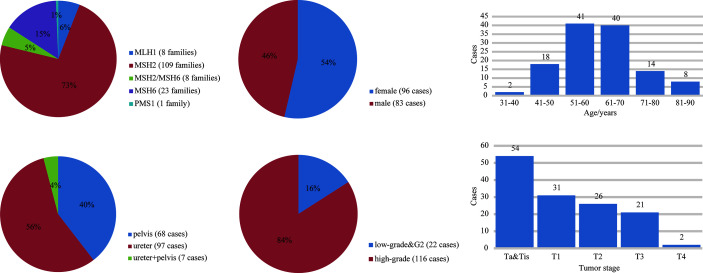
The germline variants distribution and clinicopathological features of LS-UTUC by literature review.

A total of 38 suspected LS patients underwent germline genetic testing. WES analysis showed that pathogenic/likely pathogenic (P/LP) germline variants were identified in 22 of 38 (57.9%) individuals (**Figure 2**). Five cases (5/354, 1.4%) were LS cases, including 3 cases with novel mutations. Of the 5 LS patients, 1 patient presented weak MMR protein staining, 4 patients deficient MMR protein staining, and MSI-high/low tumors were found in 4 patients (**Table 1**). Besides, patients with MMR variants of uncertain significance were identified in 7 cases, and MSI-high/low tumors occurred in 5 cases (**Table 2**). Among the 12 patients with variant of uncertain significance or P/LP variant, 5 had muscle-invasive tumors and none of them experienced lymph node metastasis or had G3 tumors (**Tables 1**, **2** and **Supplementary Table 3**). Moreover, the published literature were systematically reviewed and we found that the median age of LS-associated UTUC patients was 61 years (range 36-86 years), 73% (109 of 180) of patients had MSH2 LP/P mutation, 54% (96 of 179) of patients were female, and 36.6% (49 of 134) of patients had muscle-invasive tumors (**Supplementary Table 4** and **Figure 3**).

**Table 1:** Germline likely pathogenic/pathogenic MMR mutations in 38 patients who underwent whole exon sequencing.

ID	Gender	Age	Multifocal	Pathological feature	MMR staining	MSI staining	MMR mutation	Genotype	Mutation types	Function	History of personal history/FDR	First description
P010	female	81	yes	T3G2N0	MSH6/MSH2 -/-	high	MSH2:exon2:c.295A>T:p.Arg99*	Het	nonsense	pathogenic	colon cancer (herself, 45 years); sarcoma (herself, 70 years); bladder cancer (herself, 84 years)	this report
P075	male	41	no	T1G2N0	MSH6/MLH1 -/w	low	MSH6:exon8:c.3699dupA:p.Glu1234fs	Het	frameshift	pathogenic	none	this report
P084	male	48	no	T1G2N0	MSH6/MLH1/PMS2 w/w/w	low	MSH6:exon9:c.3880delT:p.Cys1294fs	Het	frameshift	pathogenic	Lymphoma (his father, 45 years); thyroid cancer (his sister, 40 years); thyroid cancer (his son, 19 years)	this report
P094	female	50	no	T3G2N0	MSH6/MLH1 -/-	low	PMS2:exon12:c.2012C>T:p.Thr671Met	Het	missense	likely pathogenic	none	rs587780046
P100	female	83	no	T1G2N0	MSH6 -	stable	MSH6:exon5:c.3261delC:p.Phe1088fs	Het	frameshift	pathogenic	Rectal cancer (himself, 55years)	rs267608078

MMR, mismatch repair; MSI, microsatellite instability; FDR, first-degree relative; Het, heterozygote.

**Table 2:** Germline MMR mutations with variant of uncertain significance in 38 patients who underwent whole exon sequencing.

ID	Gender	Age	Multifocal	Pathological feature	MMR staining	MSI status	Genotype	MMR mutation	Mutation types	History of personal history/FDR	First description
P003	male	55	no	T1G1N0	positive	stable	Het	MSH2:exon9:c.1470G>C:p.Lys490Asn	missense	Colon cancer (himself, 58ys; his brother, 45ys; his sister, 47ys); Rectal cancer (his brother, 53ys; his nephew, 35ys)	this report
P006	male	59	no	T1G2N0	positive	stable	Het	MSH6:exon10:c.4068_4071dupGATT:p.Lys1358fs	frameshift	Colon cancer (his uncle, 49ys; his grandmother, 45ys)	rs55740729
P009	male	56	no	T2G2N0	MSH6/MSH2 weak/-	high	Het	MSH6:exon7:c.3587A>T:p.Glu1196Val	missense	none	this report
P024	male	57	no	T3G2N0	PMS2/MSH6 weak/weak	low	Het	MSH6:exon4:c.1501C>T:p.His501Tyr	missense	none	rs779411998
P030	male	41	no	T1G2N0	MSH6/MSH2 -/-	high	Het	MSH2:exon3:c.489_494delTGGGTA	frameshift	endometrial cancer (his mother, 56ys)	this report
P066	female	57	no	T2G2N0	MSH6/MLH1 -/weak	high	Het	MSH6:exon2:c.449C>T:p.Pro150Leu	missense	none	this report
P073	male	59	no	T1G2N0	MSH6/MLH1 -/-	low	Het	MSH6:exon6:c.3529C>G:p.Leu1177Val	missense	none	rs748398941
P094	female	50	no	T3G2N0	MSH6/MLH1 -/-	low	Het	MLH1:exon8:c.649C>T:p.Arg217Cys;	missense	none	rs4986984

MMR, mismatch repair; MSI, microsatellite instability; FDR, first-degree relative; Het, heterozygote.

WES testing identified 6 cases with P/LP mutations in high-penetrance genes other than MMR genes, and 5 cases had mutations in moderate-penetrance genes only (**Figure 2**). The common P/LP variants in high- or moderate-penetrance genes were 4 in ATM, 3 in MSH6 and KIT, and 2 in APC, NF1 and DICER (**Figure 2** and **Supplementary Table 5**). Additionally, the most prevalent variants of uncertain significance in MMR, high- or moderate-penetrance genes were 8 in PKD1, 6 in FAT1, and 5 in MSH6 (**Supplementary Figure 3, 4** and **Supplementary Table 6**). Surprisingly, no LP/P or VUS mutation of TP53 gene was found in 38 patients. In total, 11 patients had germline P/LP variants in DNA damage repair (DDR) genes, and 19 patients had one or more DDR with variant of uncertain significance (**Figure 2** and **Supplementary Table 6**).

### Clinical Characteristics Associated With P/LP Variants

In 38 patients who underwent WES, 16 patients had moderate- or high-penetrance P/LP variants (**Figure 2**). About 80% (4 out of 5) of patients with multi-organic cancer had moderate- or high-penetrance P/LP variant, and 62.5% (10 out of 16) of patients with moderate- or high-penetrance P/LP variant had a history of personal second malignancy or cancer in the first-degree relatives (**Figure 2** and **Supplementary Table 5**). Both probands (P003 and P006) met the Amsterdam II criteria, but they had positive MMR protein staining and a stable microsatellite status (**Supplementary Figure 5** **)**, however, LP/P variants in MMR genes were not identified **(** **Supplementary Table 5**). Further germline mutation analysis showed that they all carried multiple other high- and moderate-penetrance LP/P variants **(** **Supplementary Table 5**). One case had one high-penetrance variant (CDH1) and 6 moderate-penetrance variants (ATM, NF1, NRAS, KIT, DICER1, and BLM), and another case had 2 high-risk variants (BRCA2 and MET) and 5 moderate-risk variants (ATM, NF1, KIT, DICER1 and JAK2). (**Supplementary Table 5**).

### MSI Analysis

MSI was significantly associated with deficient MMR protein staining (p=0.018, **Supplementary Table 2**). In 21 patients with deficient MMR protein staining, approximately 81% of tumors were harboured MSI, but only 46.7% were harboured MSI in weak MMR protein staining by IHC (**Supplementary Figure 6**). In 5 LS cases, tumors of 4 patients were harboured MSI-H/L. In 7 patients with the MMR variants of uncertain significance, 5 patients had MSI-harboring tumors. Moreover, MSI-harboring tumors were found in 76.5% (13 out of 17) of patients with personal second cancer or with cancer in first-degree relatives (**Supplementary Table 2**).

## Discussion

In this study, we roughly identified the morbidity of LS-related UTUC and carriers with LP/P germline mutations in suspected LS patients. On account of recall bias, only 3 patients met the Amsterdam II criteria in our cohort, but were not identified as LS-associated UTUC. Evidently, a series of studies identified that approximately 90% of patients with LS-related UTUC present MSI-harboring tumors (4–6, 14, 15). Consistent with previous findings, our results showed that 80% of LS cases demonstrated MSI-harboring tumors and 71.4% of patients with the MMR variants of uncertain significance present MSI-harboring lesions **(** **Table 1** **)**. In contrast, for patients with urothelial carcinoma (including pelvis, ureter and bladder tumors), Alicia Latham et al. (16) reported that only 3.6% of non-LS cases demonstrated MSI, and 37.5% of patients with MSI-harboring urothelial tumors were LS. Strict selection for MSI detection is probably the most likely reason for this discrepancy.

In our study, a total of 19% (4 out of 21) of patients with deficient MMR protein staining and 6.7% (1 out of 15) of patients with weak MMR protein staining were identified as LS-related UTUC. According to rigorous molecular diagnosis criteria, all tumor cells without any MMR protein staining could be considered a loss of MMR protein expression, which was then followed by genetic testing for LS. One patient (ID: P084) had an MSI-L-harboring tumor and weak MSH2 protein staining by IHC but was still identified as LS by genetic testing (**Table 1**). The IHC staining image of P084 is demonstrated in **Supplementary Figure 2**, which shows that the top of the tumor away from normal tissue showed deficient MSH2 protein staining, whereas the proximal tumor showed weak MMR protein staining. Although pathogenetic mutations in the MMR gene could lead to tumorigenesis in LS patients, the regulation of MMR gene expression in separate tumor areas is influenced by the temporal and spatial heterogeneity of tumor growth. Besides, it is very possible that tumor mosaicism which partly expresses MMR protein contributes to several cells with stained. As a consequence, screening LS patients by IHC staining method remains further improved.

The prevalence of LS among consecutive UTUC patients was at least 1.4% (5 out of 354) in our cohort. Previous studies found that the incidence rate of LS-related UTUC ranged from 1.4% to 21.3% (5–7, 17). In our study, 3 novel LP/P MMR mutations were found, and 3 out of 5 LS-UTUCs were carriers of the MSH6 variant, but the major variant of previous studies was MSH2 (**Table 1** and **Figure 3**). Despite relatively small sample size and single-center nature, these data imply that the primary reason leading to this difference is probably LS diagnosis pathway and geographic distribution. In fact, it has been reported that aristolochic acid intake not only damages kidney tubules, resulting in renal insufficiency, but is also confirmed to be a carcinogen leading to urothelial carcinoma, especially in Asian regions (18, 19). Although LS patients were at higher risk for colorectal cancer, endometrial cancer and other LS-related tumors over their lifetimes, 5 LS-related UTUCs in our study were alive and no metastasis occurred within a median follow-up of 45 months. The Kaplan-Meier plot showed that LS patients tended to have a favorable prognosis (**Supplementary Figure 7**). Certainly, García-Tello et al. (20) discovered that MMR gene expression was associated with a favorable prognosis and Hollande et al. (21) also reported that adjuvant chemotherapy could improve survival rate of advanced UTUC patients with hereditary-like tumors compared with those with sporadic tumors. Accordingly, good surveillance annually to prevent disease recurrence and suitable treatment for surgical management or chemotherapy would be key to acquiring a good outcome.

In addition, we surprisingly confirmed unsuspected germline mutations in high- and moderate- susceptibility genes in 16 of 38 individuals (**Figure 2**). A multicenter study performed germline analysis with a 25-gene targeting sequencing panel from 1260 individuals who had a history of LS-associated tumor, and they identified 9.0% probands with LS mutations and 5.6% probands with mutations in non-LS cancer predisposition genes (9). In addition, a single-center retrospective study investigated the proportion of young colorectal cancer cases associated with genetic predisposition, and found that approximately 20% of individuals carried one or more cancer susceptibility gene mutations (13). Our study found 5 (13.2%) cases with LS mutations and 11 (28.9%) cases with high- and moderate- penetrance gene mutations in 38 suspected LS patients. Even a more rigorous screening criteria was performed in our study, performing NGS may be of great necessity in patients with young age of onset or a history of family LS-related cancer.

As previous studies reported, Yurgelun et al. (9) found that the common LP/P high or moderate genetic susceptibility genes in suspected LS patients included BRCA1/2, ATM, CHEK2 and APC. Moreover, Carlo et al. (10) discovered the most common germline P/LP variants were BRCA1/2, APC, CHEK2, ERCC3 and ATM. In our study, there was only one patient with a BRCA2 mutation, and none carried the CHEK2 variant (**Figure 2** and **Supplementary Table 5**). Two patients (P003 and P006) who met Amsterdam criteria, whose families suffered from colorectal cancer, lung cancer and bladder cancer, were not identified as MMR mutation carriers, but they carried many other tumor-associated genes, such as BRCA2, CDH1, MET, ATM, NF1. To date, BRCA1/2 has been very well studied in breast cancer and gynecological oncology, and BRCA2 mutation carriers have been described to be significantly associated with an elevated risk for prostate cancer, colorectal cancer and urothelial carcinoma (22–25). It has been reported that CDH1 and ATM germline mutation was associated with colorectal cancer (26, 27), and MET mutation plays an important role in lung cancer and colorectal cancer development and progression (28). However, we failed to acquire the medical record of neurofibroma, NF1 germline mutation associated tumor, in their families. In addition, APC germline mutations were found in some patients who had not a second cancer and family history of cancer (**Supplementary Table 5**). Recall bias and incomplete medical examination is the potential reason leading to disagreement between genotype and phenotype correspondences.

Recently, DDR somatic and inherited mutations have been found to independently guide immune checkpoint inhibitor therapy for individuals with advanced urothelial carcinoma (29), so we analyzed the prevalence of DDR mutations in suspected LS-related UTUC patients. We found that 11 patients carried germline P/LP variants in DDR genes, and 19 patients carried one or more variants of uncertain significance (**Supplementary Tables 5** **,** **6** and **Supplementary Figure 3**). To date, the efficacy of chemotherapy for advanced/metastatic cancer in individuals with LS or MSI-H has not yet been clarified, but the efficacy of PD-1 inhibition in metastatic deficient MMR protein staining or MSI-H solid tumors has been demonstrated (30–32). Therefore, germline DDR mutation testing should actually be considered when evaluating its association with therapeutic benefit. Accordingly, whether germline variants of uncertain significance in these genes are also related to treatment and survival need further exploration.

Our research has several limitations. First, single-center and small sample retrospective studies are the main shortcomings. Clinical data concerning personal/family histories and prognosis information were retrospectively acquired by medical reports and telephone interviews, so we could not confirm its accuracy and integrity. Next, we were unable to detect MLH1 promoter hypermethylation in patients with MSI or deficient MMR protein staining, resulting in underestimation of the morbidity of LS. Finally, approximately 10% of LS-related colorectal patients showed intact MMR protein staining on IHC (2), but we only assessed genetic susceptibility gene mutations of suspected LS based on MMR protein staining or clinical criteria. As a result, we cannot fully identify potential germline variants of high-risk patients, such as in the younger patients.

Despite these limitations, our study’s main strength provides a promising direction regarding the hereditary risk assessment of suspected LS patients in the age of NGS. The advantage of such NGS diagnosis approach has been broadly discussed, and moreover, NGS testing strategies based on the phenotypes of probands have been performed in secondary analyses of germline mutation evaluation, and many potential non-LS pathogenic germline variants have been identified (9, 10, 13). **Supplementary Figure 8** provides a feasible genetic mutation analysis pathway for possible hereditary UTUC individuals. However, extensive use of NGS will undoubtedly bring about a dilemma in which increased subjects will be identified as variants of uncertain significance or other inherited variants with vague clinical significance. How patients with unexpected mutations identified by NGS are properly managed probably becomes a growingly prevalent issue for genetic clinicians.

## Conclusion

We identified approximately 11% of UTUC patients as suspected LS and at least 1.4% of patients as LS-related UTUC. In addition, in individuals with suspected LS, NGS identified many unexpected high- and moderate- penetrance mutations in genetic predisposition genes. Therefore, broader germline genetic testing, particularly NGS, could be considered to screen for cancer severity in hereditary UTUC patients.

## Data Availability Statement

The datasets presented in this study can be found in online repositories. The names of the repository/repositories and accession number(s) can be found in the article/**Supplementary Material**.

## Ethics Statement

The studies involving human participants were reviewed and approved by Peking University First Hospital Ethics Committee. Written informed consent for participation was not required for this study in accordance with the national legislation and the institutional requirements. Written informed consent was not obtained from the individual(s) for the publication of any potentially identifiable images or data included in this article.

## Author Contributions

LZ supervised all the studies. LZ, XL, YG, and SH designed the study. BG, JW, QT, and LL performed the acquisition of the data and sequencing. BG and JW analyzed and interpreted the data. BG, QT, and LL performed the statistical analysis. BG and JW drafted the manuscript. BG, DF, SH, YG, XL, and LZ revised the manuscript. WK, CT, and JL contributed material support. KY, GH, YW, and YH contributed the sample collection and clinical information. QT, LL, and YP contributed material support. YY and QH contributed administrative and technical support. All authors critically commented on and approved the final submitted version of the manuscript.

## Funding

This work was supported by the National Natural Science Foundation of China (81972380 to XL and 81772703 to YG), the National Key R&D Program of China (2019YFA09006001 to YG) and the Scientific Research Seed Fund of Peking University First Hospital (2019SF25 to QT) and Wuxi "Taihu Talents Program" Medical and Health High-level Talents Project.

## Conflict of Interest

The authors declare that the research was conducted in the absence of any commercial or financial relationships that could be construed as a potential conflict of interest.

## Publisher’s Note

All claims expressed in this article are solely those of the authors and do not necessarily represent those of their affiliated organizations, or those of the publisher, the editors and the reviewers. Any product that may be evaluated in this article, or claim that may be made by its manufacturer, is not guaranteed or endorsed by the publisher.
