# Supplementary material for: Identification of Germline Mutations in Upper Tract Urothelial Carcinoma With Suspected Lynch Syndrome
Source: Front Oncol. 2022 Mar 16;12:774202. doi: 10.3389/fonc.2022.774202 (PMC8966221; doi:10.3389/fonc.2022.774202)
Supplement: Supplementary file 1 [file DataSheet_1.docx]

**Materials and methods**

**DNA extraction**

The total genomic DNA was extracted from FFPE tissues with a commercial DNA extraction kit, the GeneRead DNA FFPE Kit (Qiagen, Germany, 180134). The quantity of the DNA samples after purification was determined with a Qubit 2.0 fluorometer (Invitrogen, USA, Q33230). Normal tissues—the renal cortex after nephroureterectomy or the normal ureter—were used for germline testing and tumor tissues with a tumor purity over 80% were used for MSI detection.

**Whole exon sequencing**

DNA library construction and exon region capture were carried out with an Agilent SureSelect Human All Exon V6 kit (Agilent, USA). In brief, after the library was built, we applied the qPCR method for accurate quantification of the library, and then we used an Agilent 2100 (Agilent, USA) to detect the insert size of the library. Finally, the Illumina sequencing platform (Illumina, USA) was used for PE150 sequencing. Preliminary processing involving base calling, read filtering, and adapter trimming was implemented following the typical Illumina analysis pipeline, and then high-quality reads were mapped to the human reference genome hg19 (http://genome.ucsc.edu/) by the BWA-MEM tool[1]. Here, Picard (https://broadinstitute.github.io/picard/) was used to remove duplicate reads. The GATK pipeline with best practice guidelines (https://software.broadinstitute.org/gatk/) was used to detect realignment at insertion/deletion sites (indels) and perform base quality score recalibration[2]. The variants were thereafter annotated using ANNOVAR software (http://annovar.openbioinformatics.org/)[3]. The mean mapping ratio was 99.93%, the median depth of coverage was 120x, and the mean bases covered on-target percentage was 99.68%.

**IHC**

In short, 4 μm thick FFPE tissue sections were stained with primary antibodies against MSH2 (Abcam, UK, ab52266, mouse monoclonal), MSH6 (Abcam, UK, ab92471, rabbit monoclonal), MLH1 (Abcam, UK, ab92312, rabbit monoclonal), and PMS2 (Abcam, UK, ab110638, rabbit monoclonal). Antigen recovery at 120℃ was performed with citrate buffer. Diaminobenzidine was applied as a color-developing agent, and hematoxylin as a redyeing agent. For the negative control, phosphate buffered saline was used to replace the specific primary antibody.

**Supplementary Figure S1-figure S7**


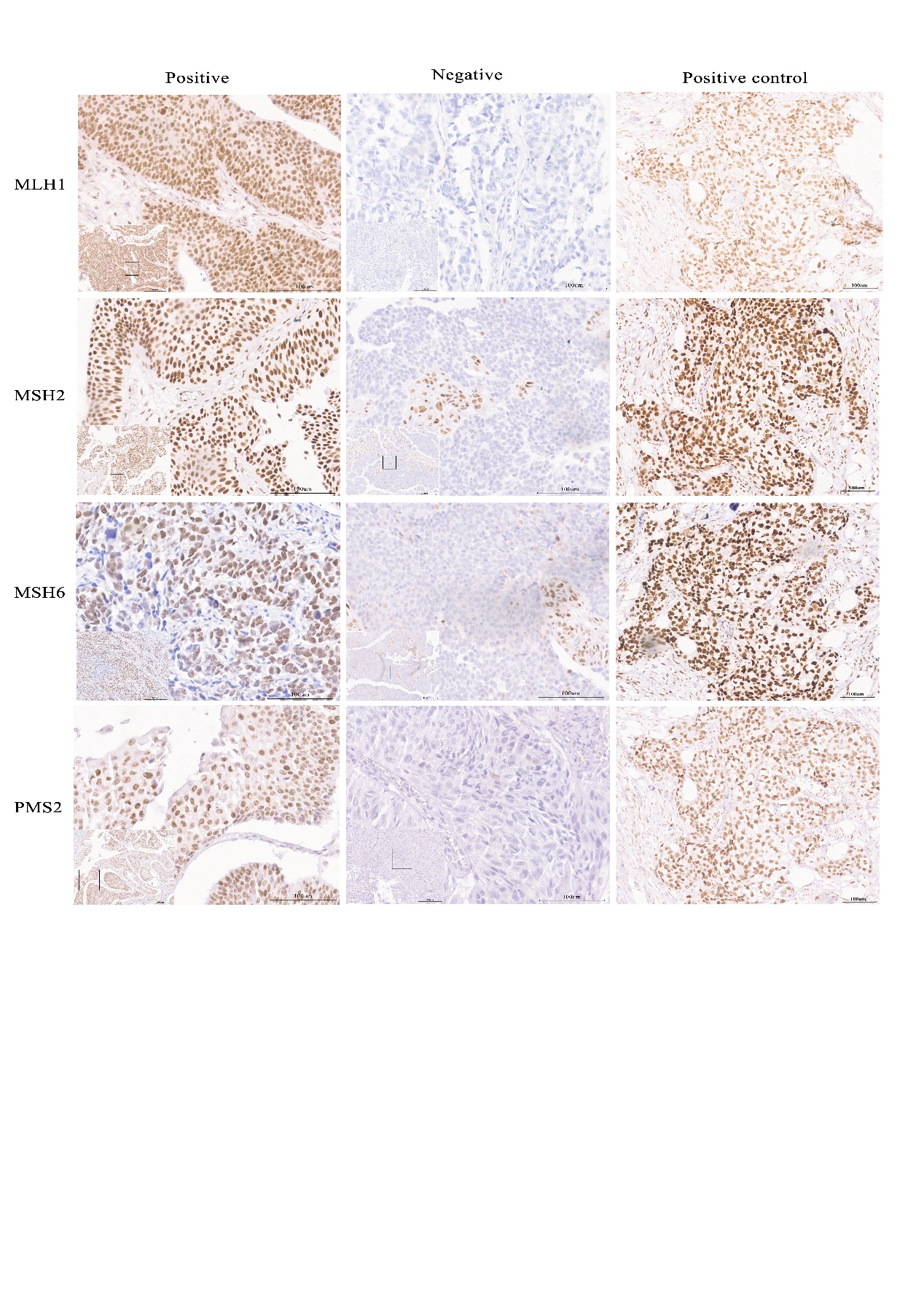


Figure S1. Expression of MMR protein in UTUC. Proficient MMR protein staining in cancer cells (left); deficient MMR protein staining in cancer cells (middle); a MMR protein proficient case with positive MMR expression served as positive control (right).


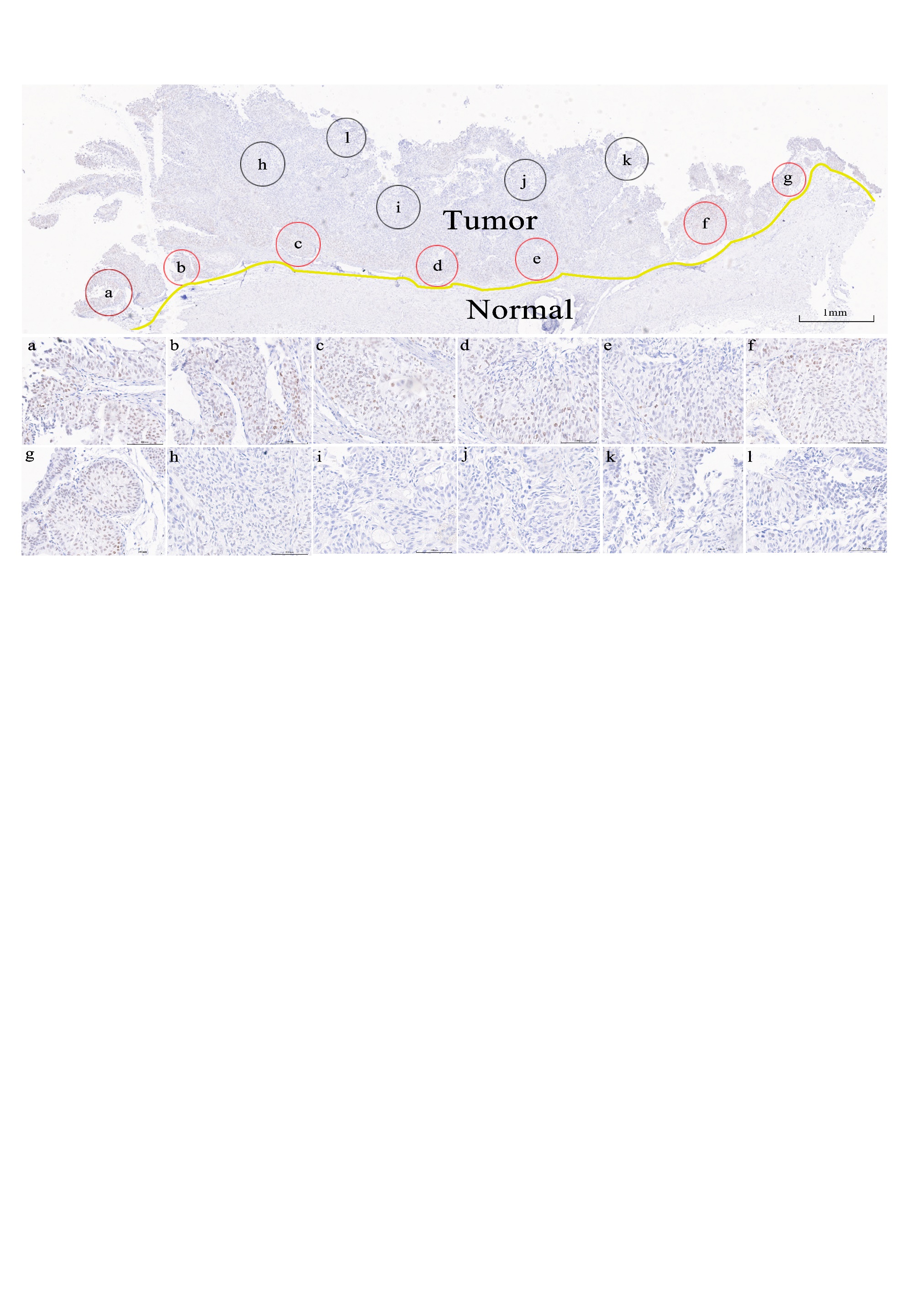


Figure S2. Staining of MSH6 protein in patient P084. The top image with a low-power field showed a consecutive tissue from tumor region to normal region and the middle as well as the bottom image with a high-power field showed MSH6 protein staining status of tissues in the circles in top image. The red cycles (a-g) demonstrated weak MSH6 protein staining; the black cycles (h-l) demonstrated absent MSH6 protein staining.


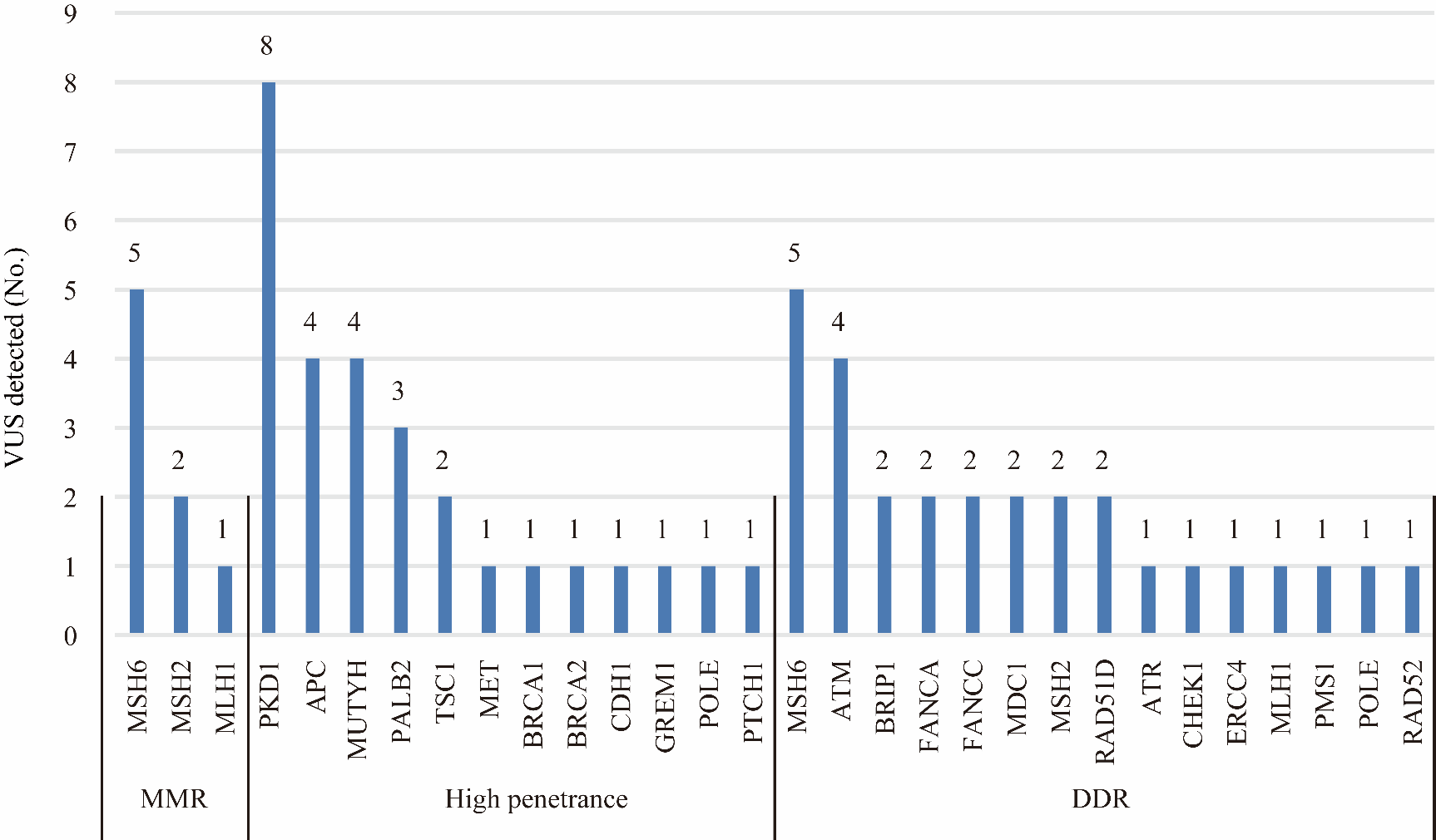


Figure S3. Number of VUS, per gene (mismatch repair genes, high penetrance genes and DNA damage repair genes), detected with a whole exon sequencing in 38 patients. VUS: variant of uncertain significance; MMR: mismatch repair; DDR: DNA damage repair genes


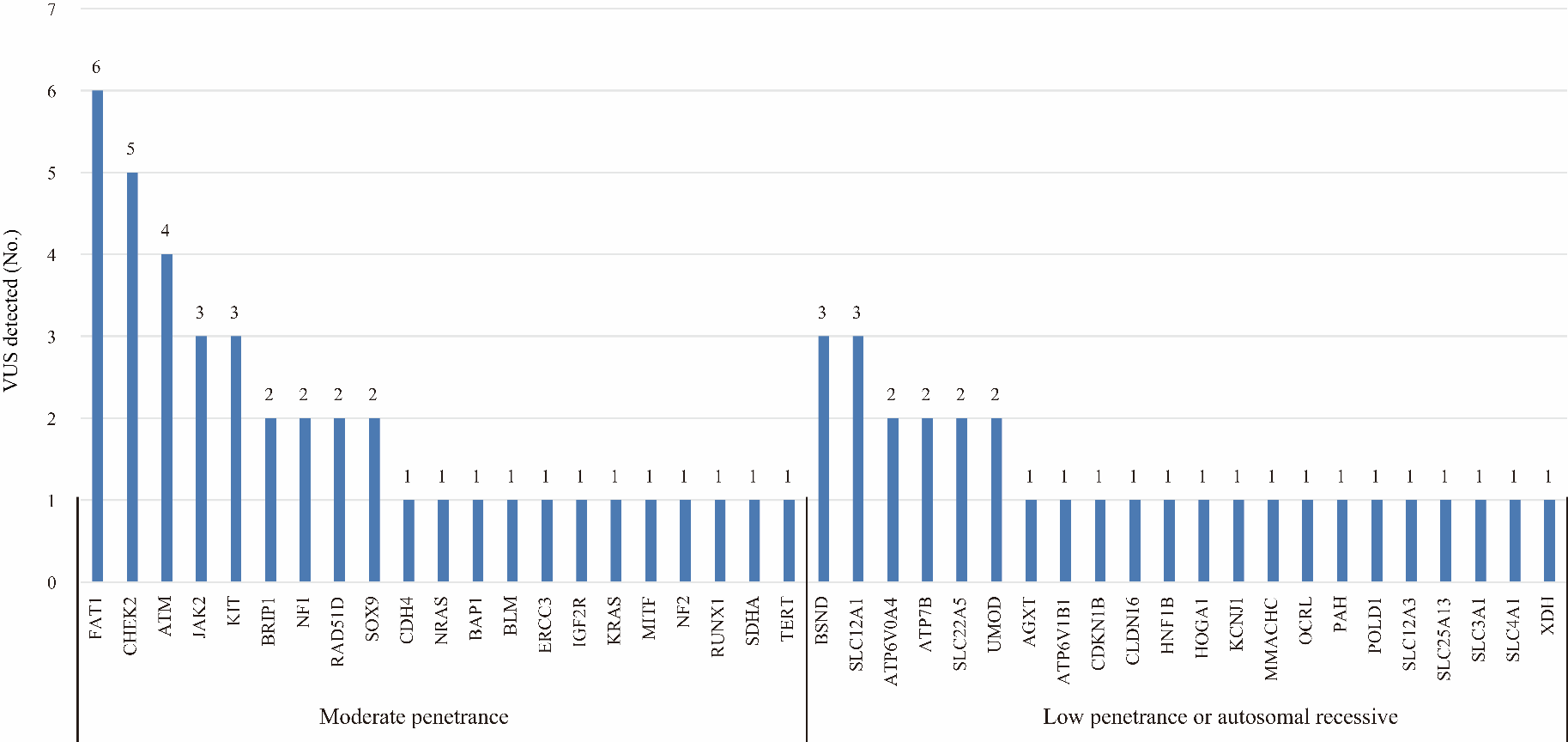


Figure S4. Number of VUS, per gene (moderate penetrance genes, low penetrance and autosomal recessive genes), detected with a whole exon sequencing in 38 patients. VUS: variant of uncertain significance





Figure S5. Pedigree of patients who met Amsterdam II criteria and were identified non-LS by genetic testing. UTUC: upper tract urothelial carcinoma; MMR: mismatch repair; MSI: microsatellite instability.


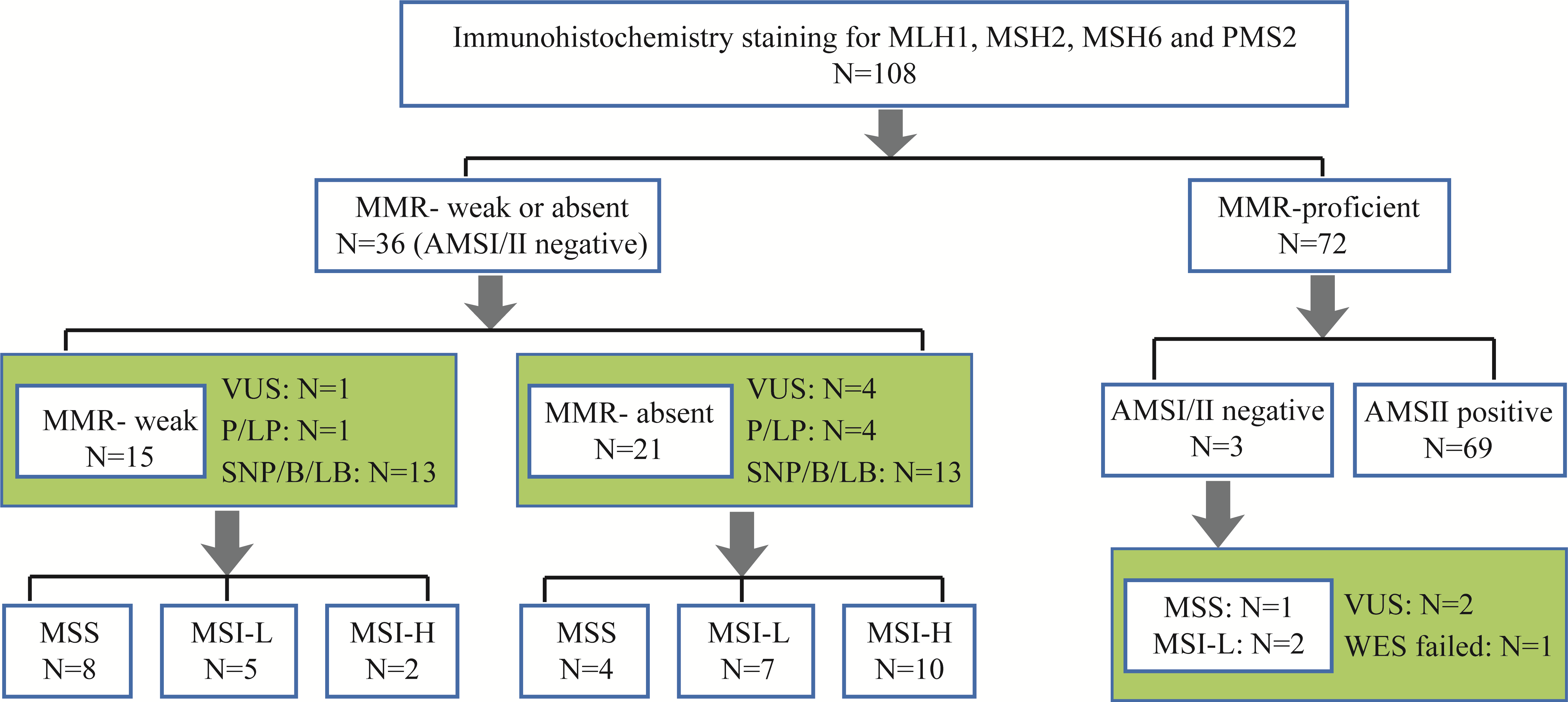


Figure S6. Summary of the clinical diagnosis criteria, immunohistochemical staining, MSI, and MMR gene mutation analyses in UTUC patients. MMR: mismatch repair; VUS: variant of uncertain significance; P/LP: pathogenic/likely pathogenic; SNP: single nucleotide polymorphism; B/LB: benign/likely benign; AMS: Amsterdam; MSS: microsatellite stable; MSI-L: microsatellite instability-low; MSI-H: microsatellite instability-high; WES: whole exon sequencing.





Figure S7. Kaplan-Meier plots for cancer specific survival, bladder recurrence-free survival and metastasis-free survival.





Figure S8. Gene screening of hereditary UTUC. UTUC: upper tract urothelial carcinoma; FDR: first degree relative; MMR: mismatch repair; IHC: immunohistochemical staining; LP: likely pathogenic

Reference

[1] Li H, Durbin R. Fast and accurate long-read alignment with Burrows-Wheeler transform. Bioinformatics. 2010 Mar 1: **26**:589-95

[2] McKenna A, Hanna M, Banks E, et al. The Genome Analysis Toolkit: a MapReduce framework for analyzing next-generation DNA sequencing data. Genome Res. 2010 Sep: **20**:1297-303

[3] Wang K, Li M, Hakonarson H. ANNOVAR: functional annotation of genetic variants from high-throughput sequencing data. Nucleic Acids Res. 2010 Sep: **38**:e164
