# Supplementary material for: Identification of Germline Mutations in Upper Tract Urothelial Carcinoma With Suspected Lynch Syndrome
Source: Front Oncol. 2022 Mar 16;12:774202. doi: 10.3389/fonc.2022.774202 (PMC8966221; doi:10.3389/fonc.2022.774202)
Supplement: Supplementary file 2 [file Table_1.docx]

Table S1 Gene list analyzed in this study.

| Grouping | Genes |
| --- | --- |
| Lynch syndrome related genes | MLH1, MSH2, MSH6, PMS2, EPCAM |
| High penetrance genes | APC, AXIN2, BMPR1A, BRCA1, BRCA2, CDH1, CDK4, CDKN2A, GREM1, MEN1, MET, MUTYH, PALB2, PKD1, PKD2, POLE, PTCH1, PTEN, RB1, RET, SDHB, SMAD4, SMARCA4, SMARCB1, STK11, TP53, TSC1, TSC2, VHL, WT1 |
| Moderate penetrance genes | ALK, ATM, BAP1, BARD1, BLM, BRIP1, CHEK2, DICER1, EGFR, ERCC2, ERCC3, FAM175A, FAT1, FH, FLCN, GATA2, HOXB12, HRAS, IGF2R, JAK2, KIT, KRAS, MAX, MITF, MRE11A, NBN, NF1, NF2, NRAS, PAX5, PDGFRA, PHOX2B, RAD50, RAD51, RAD51B, RAD51C, RAD51D, RECQL4, RUNX1, SDHA, SDHAF2, SDHC, SDHD, SMAD3, SOX9, SUFU, TERT, TGFBR1, TGFBR2, TMEM127 |
| Low penetrance or autosomal recessive related genes | AGXT, APRT, ATP6V0A4, ATP6V1B1, ATP7B, BSND, CDKN1B, CLCN5, CLCNKA, CLCNKB, CLDN16, CLDN19, CYP21A2, G6PD, GALT, GRHPR, HBA1, HBA2, HBB, HNF1B, HOGA1, HPRT1, KCNJ1, LMNA, MMACHC, MUC1, OCRL, PAH, PKHD1, POLD1, PTS, SCNN1B, SCNN1G, SLC12A1, SLC12A3, SLC22A5, SLC25A13, SLC3A1, SLC4A1, SLC7A9, UMOD, XDH |
| Other genes | AURKA, ABCC1, ABL1, AKT1, AKT3, ARID1A, ASXL1, ATR, BRAF, CARD11, CASP8, CBL, CDC73, CEBPA, CREBBP, CRLF2, CSF1R, CTNNA1, CTNNB1, CTSL1, CYLD, DNMT3A, EP300, ERBB2, ERBB3, ERBB4, EZH2, FAM123B, FBXW7, FGFR1, FGFR2, FGFR3, FIGF, FKBP9, FLT1, FLT3, FLT4, FOXL2, GALNT12, GATA1, GNA11, GNAQ, GNAS, GRIN2A, HDAC4, HNF1A, HSP90B1, IDH1, IDH2, IGF1, IGF2, IL7R, JAK3, KDM6A, KDR, KLLN, MAP2K1, MAP2K4, MPL, MSH3, MTOR, MYC, MYD88, NOS1, NOTCH1, NOTCH2, NPM1, NTN3, PARP1, PARP4, PDGFRB, PIK3C2A, PIK3CA, PIK3R1, PIK3R5, POLR3A, PPP2R1A, PRKAR1A, PRKCE, PTGS2, PTPN11, PTPRC, ROS1, SETD2, SMAD2, SMAD7, SMO, SOCS1, SRC, SYNE1, TET2, TNFAIP3, TNKS, TOP1, TRRAP, TSHR, XPO1 |
| DNA damage repair related genes | ATM, ATR, BLM, BRCA1, BRCA2, BRIP1, CHEK1, CHEK2, EPCAM, ERCC2, ERCC3, ERCC4, ERCC5, FANCA, FANCC, MDC1, MLH1, MRE11A, MSH2, MSH6, MUTYH, NBN, PALB2, PARP1, PMS1, PMS2, POLE, RAD50, RAD51, RAD51B, RAD51C, RAD51D, RAD52, RAD54L, RECQL4 |
