# Supplementary material for: Identification of Germline Mutations in Upper Tract Urothelial Carcinoma With Suspected Lynch Syndrome
Source: Front Oncol. 2022 Mar 16;12:774202. doi: 10.3389/fonc.2022.774202 (PMC8966221; doi:10.3389/fonc.2022.774202)
Supplement: Supplementary file 4 [file Table_3.docx]

Table S3. Clinicopathologic characteristics and IHC results of upper tract urothelial carcinoma patients with suspected lynch syndrome.

| Variables | | All patients | IHC result | | | Suspected lynch syndrome | | |
| --- | --- | --- | --- | --- | --- | --- | --- | --- |
|  |  |  | Present | Absent/weak | P | Negative | Positive | P |
|  |  | n=108 | n=72 | n=36 |  | n=69 | n=39 |  |
| Sex | Male | 61 | 39 | 22 | 0.493 | 36 | 25 | 0.23 |
|  | Female | 47 | 33 | 14 |  | 33 | 14 |  |
| Age | ≤55 years | 55 | 36 | 19 | 0.785 | 34 | 21 | 0.648 |
|  | >55 years | 53 | 36 | 17 |  | 35 | 18 |  |
| AA intake | Absent | 103 | 68 | 35 | 0.663 | 65 | 38 | 0.652 |
|  | Present | 5 | 4 | 1 |  | 4 | 1 |  |
| Tumor location | Left | 61 | 36 | 25 | 0.055 | 35 | 26 | 0.108 |
|  | Right | 47 | 36 | 11 |  | 34 | 13 |  |
| Tumor site | Ureter | 49 | 28 | 21 | 0.056 | 27 | 22 | 0.083 |
|  | Pelvis | 59 | 44 | 15 |  | 42 | 17 |  |
| History of bladder cancer | Negative | 100 | 64 | 36 | **0.05** | 61 | 39 | **0.049** |
|  | Positive | 8 | 8 | 0 |  | 8 | 0 |  |
| Concurrent bladder cancer | Negative | 98 | 69 | 29 | **0.015** | 66 | 32 | **0.034** |
|  | Positive | 10 | 3 | 7 |  | 3 | 7 |  |
| Contralateral UTUC | Negative | 102 | 66 | 36 | **0.176** | 63 | 39 | **0.085** |
|  | Positive | 6 | 6 | 0 |  | 6 | 0 |  |
| Tumor architecture | Papillary | 76 | 52 | 24 | 0.551 | 49 | 27 | 0.845 |
|  | Sessile | 32 | 20 | 12 |  | 20 | 12 |  |
| Tumor size | <3cm | 54 | 36 | 18 | 1 | 34 | 20 | 0.841 |
|  | ≥3cm | 54 | 36 | 18 |  | 35 | 19 |  |
| Tumor stage | Ta | 6 | 5 | 1 | 0.725 | 4 | 2 | 0.673 |
|  | T1 | 48 | 29 | 19 |  | 27 | 21 |  |
|  | T2 | 25 | 18 | 7 |  | 18 | 7 |  |
|  | T3 | 25 | 17 | 8 |  | 17 | 8 |  |
|  | T4 | 4 | 3 | 1 |  | 3 | 1 |  |
| Grade | G1 | 3 | 2 | 1 | 0.448 | 1 | 2 | 0.155 |
|  | G2 | 63 | 39 | 24 |  | 37 | 26 |  |
|  | G3 | 42 | 31 | 11 |  | 31 | 11 |  |
| Lymph node | Negative | 98 | 65 | 33 | 1 | 62 | 36 | 1 |
|  | Positive | 10 | 7 | 3 |  | 7 | 3 |  |
| Multifocal | Negative | 82 | 54 | 28 | 0.75 | 51 | 31 | 0.515 |
|  | Positive | 26 | 18 | 8 |  | 18 | 8 |  |
| Personal history of LS-related cancer | Negative | 97 | 65 | 31 | 1 | 64 | 32 | 0.199 |
|  | Positive | 11 | 7 | 5 |  | 5 | 7 |  |
| Personal history of cancer (personal UC excepted) | Negative | 95 | 63 | 31 | 1 | 62 | 32 | 0.54 |
|  | Positive | 13 | 9 | 5 |  | 7 | 7 |  |
| History of LS-related cancer in FDR | Negative | 91 | 60 | 31 | 0.709 | 60 | 31 | 0.306 |
|  | Positive | 17 | 12 | 5 |  | 9 | 8 |  |
| History of cancer in FDR | Negative | 74 | 47 | 27 | 0.305 | 47 | 27 | 0.905 |
|  | Positive | 34 | 25 | 9 |  | 22 | 12 |  |
| Differentiation | Absent | 76 | 52 | 24 | 0.551 | 49 | 27 | 0.845 |
|  | Squamous | 16 | 10 | 6 | 1 | 10 | 6 | 0.702 |
|  | Adenoid | 10 | 5 | 5 | 0.296 | 5 | 5 | 0.491 |
|  | Sarcomatoid | 13 | 8 | 5 | 0.757 | 8 | 5 | 1 |
| LVI | Absent | 91 | 64 | 27 | 0.062 | 61 | 30 | 0.116 |
|  | Present | 17 | 8 | 9 |  | 8 | 9 |  |
| Bladder recurrence | Absent | 80 | 51 | 29 | 0.277 | 49 | 31 | 0.335 |
|  | Present | 28 | 21 | 7 |  | 20 | 8 |  |
| Cancer specific death | Absent | 90 | 58 | 32 | 0.273 | 56 | 34 | 0.42 |
|  | Present | 18 | 14 | 4 |  | 13 | 5 |  |
| Metastasis | Absent | 94 | 61 | 33 | 0.378 | 59 | 35 | 0.529 |
|  | Present | 14 | 11 | 3 |  | 10 | 4 |  |

IHC: immunohistochemical staining

AA: aristolochic acid

UTUC: upper tract urothelial carcinoma

MSI: microsatellite instability

MMR: mismatch repair

LP: likely pathogenic

P: pathogenic

UC: urothelial carcinoma

FDR: first-degree relative
