# Supplementary material for: Identification of Germline Mutations in Upper Tract Urothelial Carcinoma With Suspected Lynch Syndrome
Source: Front Oncol. 2022 Mar 16;12:774202. doi: 10.3389/fonc.2022.774202 (PMC8966221; doi:10.3389/fonc.2022.774202)
Supplement: Supplementary file 5 [file Table_4.docx]

Table S4. Reports of LS-related UTUC patients underwent germline MMR detection.

| Author | Year of publication | Country | Data obtained from | Population | MSI detection | IHC detection | Mutation analysis | Frequency of LS-UTUC^#^ (149 families) |
| --- | --- | --- | --- | --- | --- | --- | --- | --- |
| Tetsuya et al.[1] | 2020 | Japan | Saitama Medical University | 164 UTUCs underwent RNU | BAT25, BAT26, D5S346, D2S123, D17S250 | MLH1, MSH2, MSH6, PMS2 | Sanger sequencing and/or MLPA | 3 cases (1.8%) |
| Donahue et al.[2] | 2019 | USA | Colon Cancer Family Registry  Memorial Sloan Kettering Cancer Center | Documented LS-associated germline mutations | Unknown | Unknown | Targeted Sequencing | 17 cases and 7 newly identified LS-UTUCs*§ |
| Shinji et al.[3] | 2018 | Japan | Toranomon Hospital | 143 serial UTUCs underwent RNU | NA | MLH1, PMS2, MSH2, MSH6. | Sanger sequencing and/or MLPA | 2 cases (1.4%) |
| Metcalfe et al.[4] | 2018 | USA | The University of Texas MD Anderson Cancer Center | 115 consecutive UTUCs | BAT25, BAT26, BAT40, D2S123, D5S346, D17S250, TGFBR20 | MLH1, MSH2, MSH6, PMS2 | Unknown germline mutation detection method | 6 cases (5.2%) *  And 6 known LS-UTUCs as a reference |
| Harper et al.[5] | 2016 | USA | Cleveland Clinic Lynch Syndrome Registry | 21 cases with a history of Lynch syndrome | NA | MLH1, MSH2, MSH6, PMS2 | Unknown | 3 cases |
| Joost et al.[6] | 2015 | Denmark, Sweden | Danish Hereditary Nonpolyposis Colorectal Cancer Registry | 55 families with LS-UTUC | BAT-25, BAT-26, NR-21, NR-24, and MONO-27 | MLH1, MSH2, MSH6 | Unknown | 84 cases from 53 families |
| Skeldon et al.[7] | 2013 | UK, USA, Canada, Netherland | Familial Gastrointestinal Cancer Registry | 321 persons with known MMR mutations | BAT25, BAT26, D2S123, D5S346, D17S250 | MLH1, MSH2, MSH6 | Sanger sequencing and/or MLPA for MLH1, MSH2, and MSH6 | 7 cases with UTUC and bladder cancer |
| Crockett et al.[8] | 2011 | Sweden | Hereditary Cancer Center in Omaha | 39 UTUCs who met Amsterdam Criteria II or had germline mutations | Unknown | Unknown | Unknown | 33 cases*§ |
| Post et al.[9] | 2010 | Netherlands | Radboud University Nijmegen Medical Centre | Carriers and first degree relatives of 95 families with a germline mutation | D2S123, D5S346, D17S250, BAT25, BAT26, BAT40 | MLH1, MSH2, MSH6 | Unknown | 9 cases from 95 families |
| ROUPRÊT et al.[10] | 2005 | France | Tenon Hospital  Pitiè-Salpètrière Hospital | 164 sporadic UTUCs | BAT25, BAT26, D2S123, D5S346, D17S250 | NA | Sanger sequencing for MSH2 | 3 cases (1.8%) |

UTUC: upper tract urothelial carcinoma

LS-UTUC: lynch syndrome related upper tract urothelial carcinoma

MLPA: multiplex ligation-dependent probe amplification

MSI: microsatellite instability

NA: not applicable

^#^ Confirmed LS-UTUC by germline mutation analysis

* Specific mutation sites information unavailable

§ Clinicopathological information of each LS-UTUC patient unobtainable

Reference

[1] Ito T, Kono K, Eguchi H, et al. Prevalence of Lynch syndrome among patients with upper urinary tract carcinoma in a Japanese hospital-based population. Jpn J Clin Oncol. 2020 Jan 24: **50**:80-8

[2] Donahu TF, Bagrodia A, Audenet F, et al. Genomic Characterization of Upper-Tract Urothelial Carcinoma in Patients With Lynch Syndrome. JCO Precis Oncol. 2018: **2018**

[3] Urakami S, Inoshita N, Oka S, et al. Clinicopathological characteristics of patients with upper urinary tract urothelial cancer with loss of immunohistochemical expression of the DNA mismatch repair proteins in universal screening. Int J Urol. 2018 Feb: **25**:151-6

[4] Metcalfe MJ, Petros FG, Rao P, et al. Universal Point of Care Testing for Lynch Syndrome in Patients with Upper Tract Urothelial Carcinoma. J Urol. 2018 Jan: **199**:60-5

[5] Harper HL, McKenney JK, Heald B, et al. Upper tract urothelial carcinomas: frequency of association with mismatch repair protein loss and lynch syndrome. Mod Pathol. 2017 Jan: **30**:146-56

[6] Joost P, Therkildsen C, Dominguez-Valentin M, Jonsson M, Nilbert M. Urinary Tract Cancer in Lynch Syndrome; Increased Risk in Carriers of MSH2 Mutations. Urology. 2015 Dec: **86**:1212-7

[7] Skeldon SC, Semotiuk K, Aronson M, et al. Patients with Lynch syndrome mismatch repair gene mutations are at higher risk for not only upper tract urothelial cancer but also bladder cancer. Eur Urol. 2013 Feb: **63**:379-85

[8] Crockett DG, Wagner DG, Holmang S, Johansson SL, Lynch HT. Upper urinary tract carcinoma in Lynch syndrome cases. J Urol. 2011 May: **185**:1627-30

[9] van der Post RS, Kiemeney LA, Ligtenberg MJ, et al. Risk of urothelial bladder cancer in Lynch syndrome is increased, in particular among MSH2 mutation carriers. J Med Genet. 2010 Jul: **47**:464-70

[10] Roupret M, Coulet F, Azzouzi AR, Fromont G, Cussenot O. Accuracy of the routine detection of mutation in mismatch repair genes in patients with susceptibility to hereditary upper urinary tract transitional cell carcinoma. BJU Int. 2005 Jul: **96**:149-51
